# Evaluation of Local Tumor Outcomes Following Microwave Ablation of Colorectal Liver Metastases Using CT Imaging: A Comparison of Visual versus Quantitative Methods

**DOI:** 10.1148/rycan.230147

**Published:** 2025-01-24

**Authors:** Joshua D. Shur, Nuria Porta, Leila Kafaei, Laura Pendower, James McCall, Nasir Khan, Wim Oyen, Dow-Mu Koh, Edward Johnston

**Affiliations:** From the Department of Radiology, Royal Marsden Hospital NHS Foundation Trust, 203 Fulham Road, London SW3 6JJ, England (J.D.S., L.K., L.P., J.M., N.K., D.M.K., E.J.); Institute of Cancer Research, London, England (N.P., D.M.K.); and Department of Radiology and Nuclear Medicine, Rijnstate Hospital, Arnhem, the Netherlands (W.O.).

**Keywords:** Interventional-Body, Liver, Neoplasms, Ablation Techniques

## Abstract

**Purpose:**

To compare visual versus quantitative ablation confirmation for identifying local tumor progression and residual tumor following microwave ablation (MWA) of colorectal liver metastases (CRLM).

**Materials and Methods:**

This retrospective study included patients undergoing MWA of CRLM from October 2014 to February 2018. Two independent readers visually assessed pre- and postprocedure images and semiquantitatively scored for incomplete ablation, using a six-point Likert scale, and extracted quantitative imaging metrics of minimal ablative margin (MAM) and percentage of tumor outside of the ablation zone, using both rigid and deformable registration. Diagnostic accuracy and intra- and interobserver agreement were assessed.

**Results:**

The study included 60 patients (median age, 71 years [IQR, 60–74.5 years]; 38 male) with 97 tumors with a median diameter of 1.3 cm (IQR, 1.0–1.8 cm). Median follow-up time was 749 days (IQR, 330–1519 days). Median time to complete rigid and deformable workflows was 3.0 minutes (IQR, 3.0–3.2 minutes) and 14.0 minutes (IQR,13.9–14.4 minutes), respectively. MAM with deformable registration had the highest intra- and interobserver agreement, with Gwet AC1 of 0.92 and 0.67, respectively, significantly higher than interobserver agreement of visual assessment (Gwet AC1, 0.18; *P* < .0001). Overall, quantitative methods using MAM had generally higher sensitivity, of up to 95.6%, than visual methods (67.3%, *P* < .001), at a cost of lower specificity (40% vs 71.1%, *P* < .001), using deformable image registration.

**Conclusion:**

Quantitative ablation margin metrics provide more reliable assessment of outcomes than visual comparison using pre- and postprocedure diagnostic images following MWA of CRLM.

**Keywords:** Interventional-Body, Liver, Neoplasms, Ablation Techniques

*Supplemental material is available for this article*.

Published under a CC BY 4.0 license.

## Graphical abstract

**Figure:**
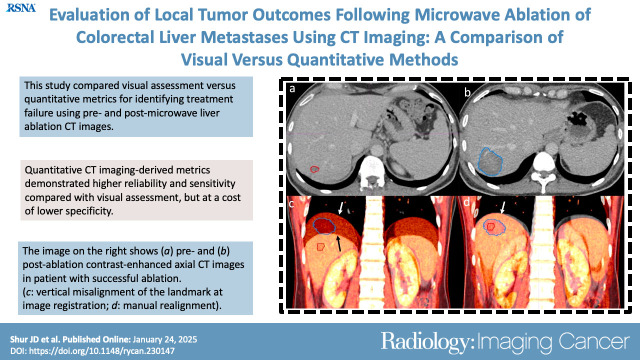

**Summary**

Quantitative analysis of pre- and postprocedure CT images more reliably identified residual tumor and local tumor progression compared with visual assessment in patients who underwent microwave ablation of colorectal liver metastases.

**Key Points**

■ Visual assessment of residual tumor and local tumor progression on pre- and postprocedure CT images in patients who underwent microwave ablation of colorectal liver metastases was unreliable (Gwet AC1, 0.18 for interobserver reproducibility).
■ Quantitative metrics of ablation success were more reliable than visual assessment, with minimal ablative margin with deformable registration achieving the highest intra- and interobserver agreement (Gwet AC1) of 0.92 and 0.67, respectively (*P* < .001 for interobserver agreement).
■ Quantitative methods using MAM had generally higher sensitivity, of up to 95.6%, than visual methods (67.3%, *P* < .001), at a cost of lower specificity (40% vs 71.1%, *P* < .001), using deformable image registration.

## Introduction

Image-guided thermal ablation is recommended by international guidelines (1) for the treatment of colorectal liver metastases (CRLM) (2,3) because thermal ablation improves survival (4) compared with chemotherapy alone.

The *ablation margin* refers to the region of normal tissue around the metastasis that should be treated to minimize the chance of later recurrence (5). Assessment of the ablation margin is crucial because clear margins provide the best chance of local tumor control (6), and a minimal ablative margin (MAM) greater than 5 mm is an independent predictor of local tumor progression (LTP) (7), which may necessitate reablation.

Technical success of complete ablation is conventionally assessed using side-by-side visual comparison of pre- and postablation CT imaging. However, visual assessment has high inter- and intrareader variability (8), such that experienced radiologists misinterpret up to 44% of cases (9). Better techniques that improve assessment of ablation margins are therefore needed.

Software-assisted ablation confirmation can be used to plan, perform, and/or evaluate treatment success (10,11). Here, pre- and postablation images are used to extract quantitative measurements describing the relationship between tumors and ablation zones for more accurate assessment of ablative margins. While such methods are in limited clinical use in the immediate postablation setting (12,13), their utility in the follow-up setting (ie, at later time points after ablation) remains uncertain. Such applications may be needed as contrast-enhanced pre- and postprocedural diagnostic CT imaging is routinely performed, whereas intraprocedural contrast-enhanced CT imaging is not always performed (14) or may not be available in case of US-guided ablation.

Software-assisted ablation confirmation can be performed with either rigid or nonrigid (deformable) methods (15). With rigid ablation confirmation, images are overlaid and aligned with translational or rotational transformations performed cognitively or computationally. However, changes in shape (eg, liver deformation due to positioning, surgery, and inspiratory effort) between the two image sets are not accounted for by this method. Nonrigid methods aim to account for changes in organ shape by deforming image sets with respect to each other (16). These methods are generally more complex and computationally intensive but are potentially more reproducible, particularly in the follow-up setting where patients are self-ventilating and scans are acquired at different phases of inspiration.

To our knowledge, there are few reports using these approaches together with pre- and postprocedure imaging for patients with CRLM undergoing ablative treatments and none that compare different ablation confirmation methods.

The imaging biomarker roadmap offers a framework for validation of putative quantitative imaging biomarkers and recommends technical validation (repeatability and reproducibility) followed by clinical validation (17).

The primary objective of this study was to assess and compare the performance of quantitative imaging metrics derived from rigid and deformable ablation confirmation using pre- and postprocedure diagnostic imaging, as compared with standard visual assessment, to detect residual tumor or LTP in patients undergoing microwave ablation (MWA) of CRLM. The secondary objective was to evaluate and compare intra- and interobserver agreement.

## Materials and Methods

This retrospective study was approved by the institutional research ethics committee, and informed consent was waived for included patients. MIM Maestro (version 7.2.9; MIM Software) was used in this study under a collaboration agreement with the same company. No study-specific funding was received. One of the authors (E.J.) declares travel reimbursement and subsistence to attend an ablation meeting. The authors had full control of the data and the information submitted for publication.

### Patients

**Figure 1:**
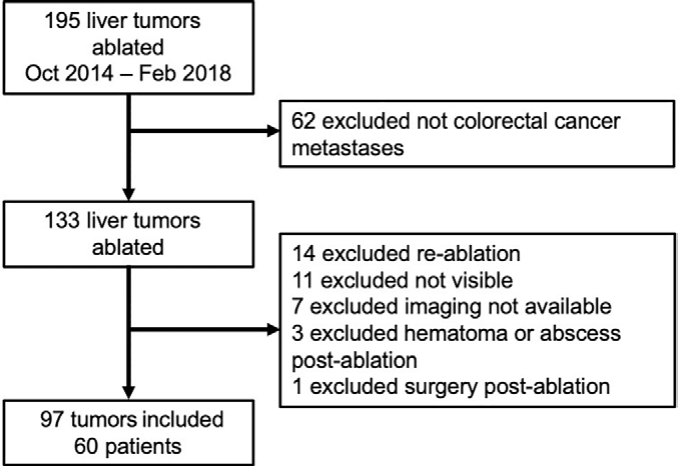
Flowchart outlines inclusion and exclusion criteria for final study cohort.

Figure 1 shows the inclusion and exclusion criteria for patient selection. Patients meeting the following inclusion criteria were included in the study: (*a*) consecutive patients who underwent MWA of the liver for treatment of CRLM from October 2014 to February 2018 (to allow sufficient time for tumors to recur, usually within 2 years), (*b*) available pre- and postprocedure portal venous phase contrast-enhanced CT imaging (defined as the preprocedural diagnostic CT imaging prior to ablation and during routine follow-up after ablation), and (*c*) imaging and clinical follow-up of at least 3 months to assess for local tumor residuum or progression. The exclusion criteria were noncolorectal cancer liver metastases, retreatment of previously ablated tumors, and severe artifact precluding accurate tumor or ablation zone segmentation (eg, hematoma or abscess).

Included tumors were confined to CRLM, as biology will differ with other tumor types and may impact the ablation zone volume necessary to achieve local control. The role of thermal ablation with CRLM is also more established compared with other secondary liver tumors. Criteria for selecting tumors for ablation were based upon international guidelines (18). Individual treatment decisions were decided following discussion at the specialist tumor board attended by oncologists, hepatobiliary surgeons, and diagnostic and interventional radiologists.

After initial postablation imaging at 3–5 weeks, surveillance was conducted with multimodality imaging using a combination of CT, MRI, and PET/CT every 3 months. A fellowship-trained attending abdominal radiologist (J.D.S.) with 11 years’ experience, with expertise in liver tumor imaging, assessed the suitability of images for inclusion in the study, blinded to other imaging such as MRI or PET/CT and clinical and follow-up data. Unenhanced intraprocedural CT scans were not used, as it was not possible to accurately define the tumor and ablation zone without the use of intravenous contrast media.

### Ablation Procedures

Procedures were performed under general anesthesia by three consultant interventional radiologists (including N.K. and J.M.) (with >10 years of experience in liver MWA) using CT guidance (Definition Edge; Siemens Healthineers), sometimes in combination with US. Spiral CT images were acquired in the axial plane without intravenous contrast media, using a 3-mm section thickness and 3-mm interval. Tumor ablation was performed using one of two systems (Solero, Angiodynamics or NeuWave, J&J), and antenna position was manipulated manually, with monitoring using intermittent low-dose sequential acquisitions (six sections, 2.4-mm section thickness, 2.4-mm section interval) until placement was judged as satisfactory for ablation.

### Image Acquisition

Preprocedural CT images were acquired before (typically 6 weeks) ablation and after (typically 6 weeks) ablation. CT scans of the abdomen and pelvis were performed with 128-section CT scanners (SOMATOM Definition Edge and SOMATOM Definition Flash; Siemens Healthineers) using iodine-based intravenous contrast medium (3–5 mL/sec, total amount of 90–150 mL, followed by a bolus injection of 30 mL of saline) in the portal venous phase with a fixed 70-second delay. Images were reconstructed at 3-mm section thickness.

Anonymized images were transferred to image processing software (MIM Maestro) for visual and quantitative assessment. MIM Maestro is a flexible imaging processing software that was adapted to create a standard ablation confirmation workflow.

### Image Analysis

***Visual assessment.—*** Two radiologists with expertise in abdominal (J.D.S.) and interventional radiology (E.J.), each with 11 years of experience, with specific expertise in liver tumor imaging, independently performed visual assessment of pre- and postablation CT studies, blinded to other imaging such as MRI or PET/CT and clinical and follow-up data. Each ablation zone was semiquantitatively scored for the perceived chances of incomplete ablation, using a six-point Likert scale (clear residual tumor, incomplete tumor coverage, probable incomplete tumor coverage, probable complete tumor coverage, successful ablation with a safety margin, and definite successful ablation with at least 5-mm safety margin) (9).

***Quantitative assessment.—*** The same two radiologists performed the quantitative analysis–image registration–ablation confirmation workflow independently. After a 1-month washout period between measurements, one of the radiologists (J.D.S.) repeated the analysis for calculation of intraobserver agreement. The total time of completion of the entire quantitative analysis workflow (using rigid and deformable registration) was recorded on a per-tumor basis.

***Rigid ablation confirmation.—*** Rigid ablation confirmation was performed on the pre- and postablation images, using MIM Maestro software. A customized workflow was implemented, which is described in detail in Appendix S1 and summarized below.

The target tumor was manually contoured on the preablation axial CT image to form a three-dimensional volume of interest. The corresponding ablation zone was then contoured in the same fashion on the postablation CT image. Image fusion of pre- and postablation scans was performed with manual corrections to adjust for misalignment at the initial fusion step, blinded to the contoured volumes, concentrating on accurate fusion of local landmarks (such as liver edge, major veins and arteries, etc) close to the ablated tumor. Box-based alignment (19) was used to optimize registration.

**Figure 2:**
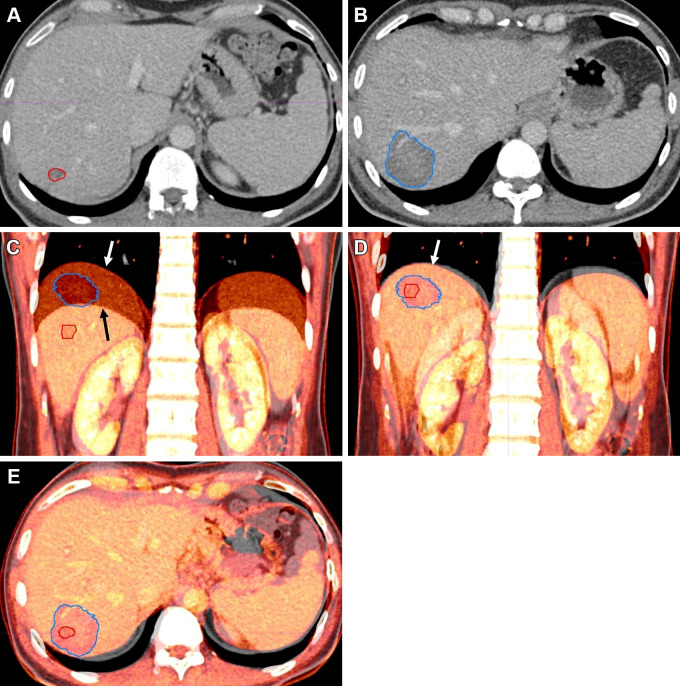
Example case shows successful ablation without evidence of local tumor progression 75 months after ablation. Contrast-enhanced axial plane CT images in a 45-year-old male patient. Tumor (red) outlined on **(A)** preablation CT image and **(B)** ablation zone (blue) on postablation CT image. **(C)** Initial fusion step shows vertical misalignment of the landmark (right hemidiaphragm, white and black arrows). **(D)** Manual alignment of right hemidiaphragm is performed (white arrow). **(E)** Box-based alignment performed over tumor (red outline in **C–E**) and ablation zone (blue outline in **C–E**), with final alignment. Percentage unablated tumor was 0% and 2.28% for measurement 1 and 2, respectively, from a single reader. At visual assessment, ablation success was scored as incomplete tumor coverage and successful ablation for readers 1 and 2, respectively.

**Figure 3:**
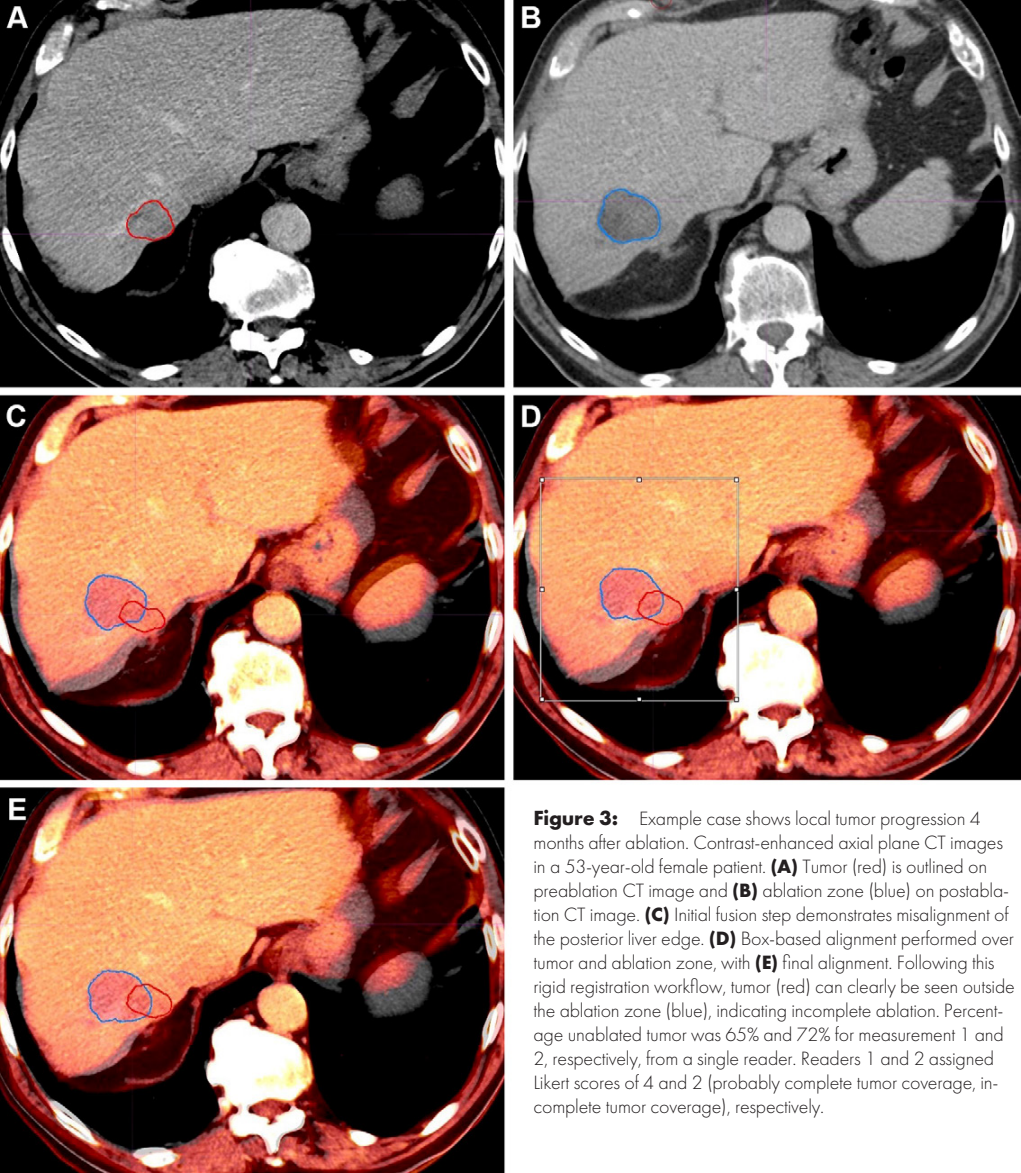
Example case shows local tumor progression 4 months after ablation. Contrast-enhanced axial plane CT images in a 53-year-old female patient. **(A)** Tumor (red) is outlined on preablation CT image and **(B)** ablation zone (blue) on postablation CT image. **(C)** Initial fusion step demonstrates misalignment of the posterior liver edge. **(D)** Box-based alignment performed over tumor and ablation zone, with **(E)** final alignment. Following this rigid registration workflow, tumor (red) can clearly be seen outside the ablation zone (blue), indicating incomplete ablation. Percentage unablated tumor was 65% and 72% for measurement 1 and 2, respectively, from a single reader. Readers 1 and 2 assigned Likert scores of 4 and 2 (probably complete tumor coverage, incomplete tumor coverage), respectively.

The rigid ablation confirmation workflow is shown in Figure S1, and example cases with LTP and the reference standard of no LTP are illustrated in Figures 2 and 3, respectively. For ablation of multiple tumors during the same session, the confirmation workflow was performed separately for each tumor.

Two metrics were then extracted: (*a*) MAM, defined as the minimum Hausdorff distance (20) between the tumor and ablation zone (measured in millimeters) and (*b*) percentage tumor volume outside of the ablation zone (%OAZ), calculated by dividing the tumor volume outside the ablation zone by the total tumor volume and multiplying by 100.

***Deformable ablation confirmation.—*** The deformable ablation confirmation workflow was identical to that described above except for the addition of an artificial intelligence (AI) algorithm (Contour Protégé AI; MIM Software) to contour the liver. These contours were used for contour-based deformable ablation confirmation. A customized workflow was implemented, summarized below, with further detail in Appendix S1. In total, this ablation confirmation method required nine steps versus five for the rigid method.

**Figure 4:**
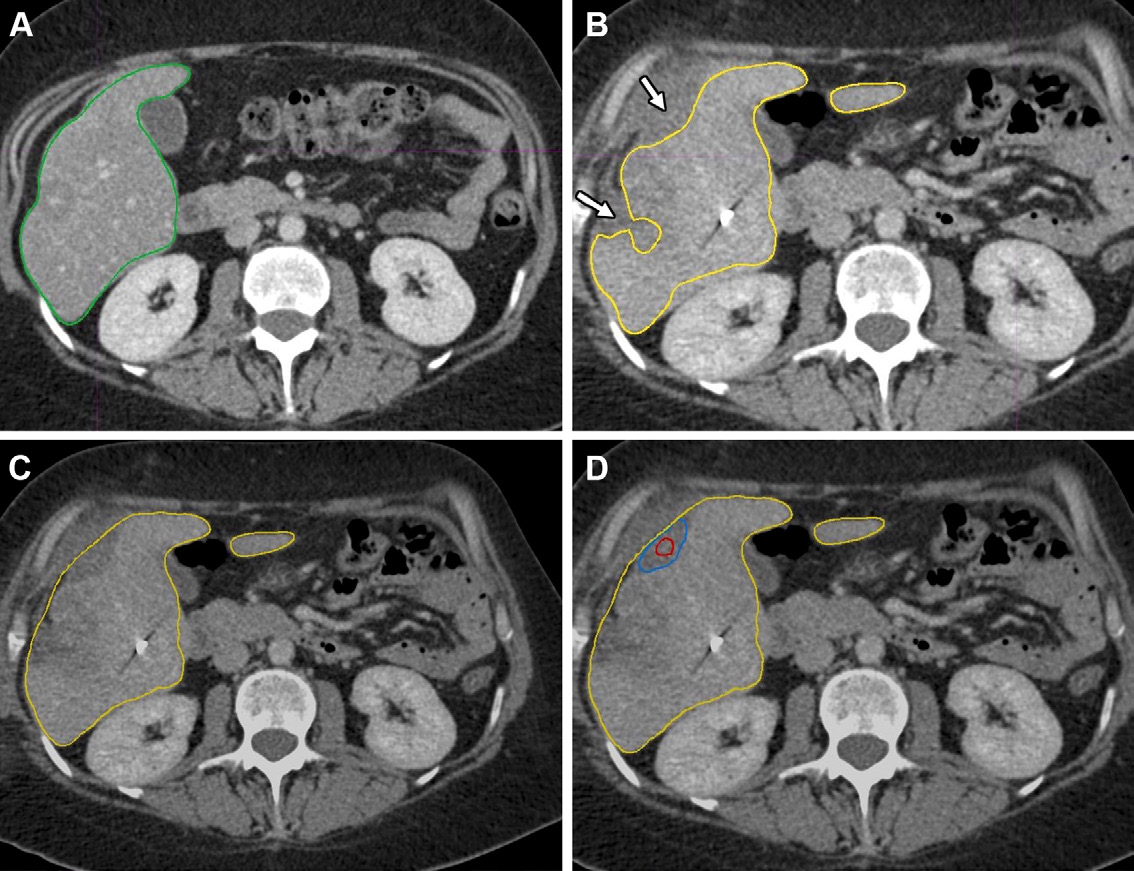
Example case using the deformable registration method. Contrast-enhanced axial plane CT images in a 63-year-old male patient. Initial contouring of tumor and ablation zone not shown. **(A)** Liver on preablation scan outlined (green) using AI-assisted segmentation. **(B–D)** On postablation scan, manual adjustments were required to correct for unsegmented liver (arrows, **B**) following AI-assisted segmentation. Yellow outline indicates liver contour on postablation scan. **(C)** Final liver contour is illustrated. Manual alignment was performed as per rigid workflow (not shown) before final deformable registration step mapping postablation liver and ablation zone contour to preablation contours. **(D)** Final overlap of tumor (red) and ablation zone (blue) is demonstrated. AI = artificial intelligence.

Initially, the AI algorithm was used to automatically segment the whole liver on pre- and postablation images, with manual corrections as needed (Figs 4 and S2). The tumor and ablation zone were then manually segmented as for rigid registration.

After box-based alignment, the liver and ablation zone from the postablation scan were mapped onto the preablation liver contour, using contour-based deformable registration. This step iteratively minimizes the difference in signed distance from the surfaces of contour pairs between the two images. AI-assisted segmentation was performed using a personal computer running Windows 10 (Microsoft) with 11th-generation Intel Core i7 with 2.5 GHz and 32 GB RAM and NVIDIA RTX 3070 laptop GPU. The approximate time for organ segmentation (including liver and lungs) was 2–3 minutes.

### Outcomes and Reference Standard

The primary outcomes were residual tumor and LTP. Residual tumor was defined as visible tumor at the edge of the ablation zone at initial follow-up imaging (5). LTP was defined as the appearance of new tumor foci at the edge of the ablation zone after at least one cross-sectional imaging study documented adequate ablation (5). When multiple new sites of disease were encountered, either in contact with the ablation zone or elsewhere in the liver, this was interpreted as systemic disease progression rather than LTP.

The reference standard for incomplete ablation was the presence of either LTP or residual tumor following review of multimodality surveillance imaging including MRI and PET/CT performed every 3 months, carcinoembryonic antigen levels, and multidisciplinary tumor board discussions. The reference standard was independently classified by the same two attending radiologists (E.J. and J.D.S.). Where there was disagreement, consensus was reached by independent scoring from a third attending interventional radiologist with expertise in liver tumor ablation. This was required in 15 of 97 (15%) cases.

Primary and secondary efficacies were defined as the percentage of tumors successfully treated at the first and second ablation session respectively, without evidence of residual tumor at follow-up imaging.

### Statistical Analysis

Medians and IQRs are reported for continuous variables. Categorical variables are summarized by frequencies and percentages.

For both readers, the diagnostic performance of semiquantitatively scored visual methods (1–6) and quantitative assessment methods (MAM and %OAZ using rigid and deformable registration) was assessed versus the reference standard. Area under the receiver operating characteristic curve (AUC) metrics were calculated. Visual and quantitative methods were compared by applying a specificity threshold of 70% (the approximate performance of visual assessment when Likert scales were binarized into groups of 1–3 and 4–6).

Sample size in this retrospective study was determined by the availability of images within the designated period. The 95% CIs were calculated for all diagnostic metrics, using a bootstrapping method. As patients had multiple tumors, 95% CIs were estimated from 1000 bootstrap samples. The bootstrapping procedure involved repeatedly randomly selecting patients with replacement from the data and calculating the outcome of interest for that sample. This process was repeated 1000 times for each analysis. Bootstrapping was used to calculate the CI for outcome statistics, whereas estimates were calculated from raw data. CIs and *P* values were calculated from the distribution of bootstrap samples. To account for repeated measurements, bootstrap samples were constructed by randomly selecting patients rather than tumors.

We conducted post hoc power calculations to evaluate the ability of our study to detect significant differences in sensitivity between visual assessment and quantitative metrics (%OAZ rigid, %OAZ deformable, MAM rigid, MAM deformable) for both readers. Using the methods described by Obuchowski et al (21), and assuming a correlation of 0.5 between AUC values from different methods, the observed sample size of 97 would have a 77% power to show a difference in AUC of 0.15 between methods (based on assumed AUC values of 0.60 and 0.75). The same sample size would have a power of 95% to show a difference in AUC of 0.20 between methods (based on assumed AUC values of 0.60 and 0.80).

Intra- and interobserver agreement were calculated using Gwet AC1 and intraclass correlation coefficient (ICC), interpreted using the following scale: 0.81–1.00, almost perfect agreement; 0.61–0.80, substantial agreement; 0.41–0.60, moderate agreement; 0.21–0.40, fair agreement; 0.01–0.20, slight agreement; 0.00 and below, no agreement or worse than random (22). Difference in interobserver agreement was assessed using 1000 bootstrapping samples to calculate a *P* value between visual assessment and the best performing quantitative imaging metric and deformable versus nondeformable registration.

Statistical analysis was performed using Stata (version 15.1).

## Results

### Patient Demographics

Sixty patients (median age, 71 years [IQR, 60–74.5 years]; 38 male) with 97 tumors were included in the study. Patient demographics and summary outcome data are outlined in Table 1. Fifty-eight of 97 (60%) ablated tumors were synchronous. Two patients did not receive systemic chemotherapy prior to ablation.

**Table 1:**
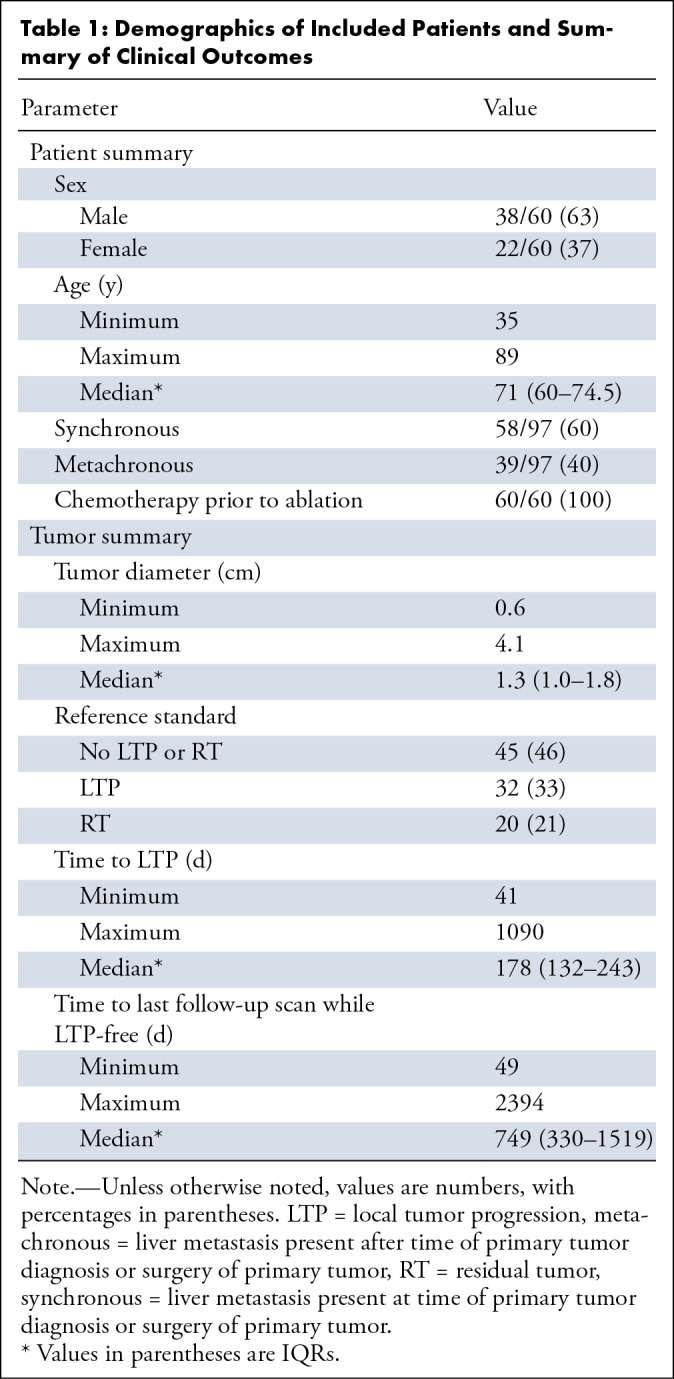
Demographics of Included Patients and Summary of Clinical Outcomes

### Imaging and Outcome Data

The median time interval between pre- and postablation CT scans was 88 days (IQR, 59–107 days). Of 97 ablated tumors, 45 of 97 tumors (46%) did not recur and had a median follow-up time of 749 days (IQR, 330–1519 days). Most tumors, 33 of 45 (73%), that did not recur had imaging follow-up of at least 1 year. Of 97 ablated tumors, 35 (36%) were peripheral or adjacent to a vessel.

According to the reference standard, 33% (32 of 97) developed LTP and 21% (20 of 97) residual tumor, with a median time to LTP of 178 days. Examples of successful and unsuccessful ablations are outlined in Figures 2 and 3. For the cases without residual tumor, the LTP-free survival rate was 64% (49 of 77) at 12 months and 62% (48 of 77) at 24 months. For those that recurred, most (28 of 32, 88%) recurred within 1 year of ablation. Primary and secondary efficacies were 46% (45 of 97) and 52% (50 of 97), respectively.

Ablation margin results using visual assessment, rigid and deformable registration, and clinical outcomes are presented in Table S1.

### Visual Assessment

Using visual assessment, AUC for reader 1 was 0.69 (95% CI: 0.58, 0.78) and for reader 2, 0.76 (95% CI: 0.64, 0.84) (Table 2).

**Table 2:**
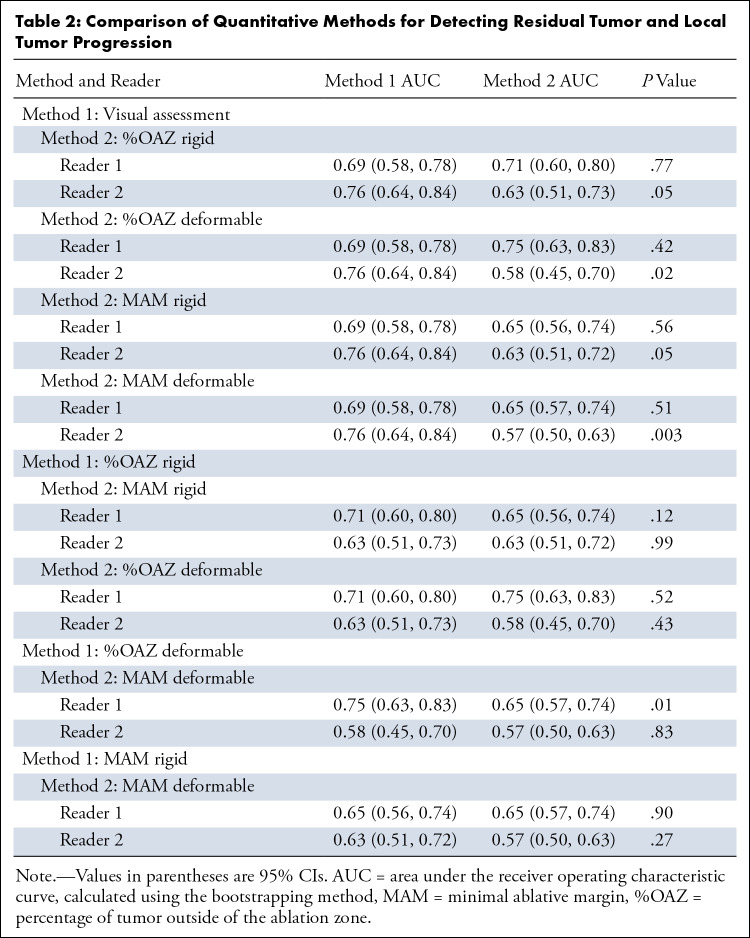
Comparison of Quantitative Methods for Detecting Residual Tumor and Local Tumor Progression

### Quantitative Assessment

The median time to complete the entire rigid and deformable workflows was 3 minutes (IQR, 3.0–3.2 minutes) and 14 minutes (IQR, 13.9–14.4 minutes) per tumor, respectively. The time penalty incurred with the deformable workflow was predominantly driven by the AI-assisted liver segmentation step requiring higher computational complexity, with the need for manual correction of liver contours contributing to a lesser extent. An example of the deformable registration method is outlined in Figure 4.

### Visual and Quantitative Assessment

AUC values ranged between 0.57 (95% CI: 0.50, 0.63) and 0.76 (95% CI: 0.64, 0.84) (Table 2). For reader 2, pairwise comparison between visual and quantitative AUC demonstrated significant differences with %OAZ deformable (0.76 [95% CI: 0.64, 0.84] vs 0.58 [95% CI: 0.45, 0.70], respectively; *P* = .02) and MAM deformable (0.76 [95% CI: 0.64, 0.84] vs 0.57 [95% CI: 0.50, 0.63], respectively; *P* = .003). There was no evidence of significant differences between pairwise comparisons of AUC between quantitative metrics, apart from a higher performance of 0.75 (95% CI: 0.63, 0.83) versus 0.65 (95% CI: 0.57, 0.74) for the first reader (*P* = .01) with %OAZ deformable.

Quantitative methods had a higher sensitivity and lower specificity than visual assessment across most comparisons (Table 3). For example, reader 1 had a sensitivity of 53.8% (95% CI: 38.5, 65.4) with 73.3% (95% CI: 57.8, 84.4) specificity using visual assessment, while MAM with deformable registration had a 90.4% sensitivity (95% CI: 82.3, 98.1) at 40.0% specificity (95% CI: 24.4, 53.3) (*P* < .001). Reader 2 had a sensitivity of 67.3% (95% CI: 51.9, 76.9) and 71.1% specificity (95% CI: 55.6, 82.2) using visual assessment, while MAM with deformable registration had a sensitivity of 95.6% (95% CI: 85.4, 100) at 40.0% specificity (95% CI: 24.4, 53.3) (*P* < .001).

**Table 3:**
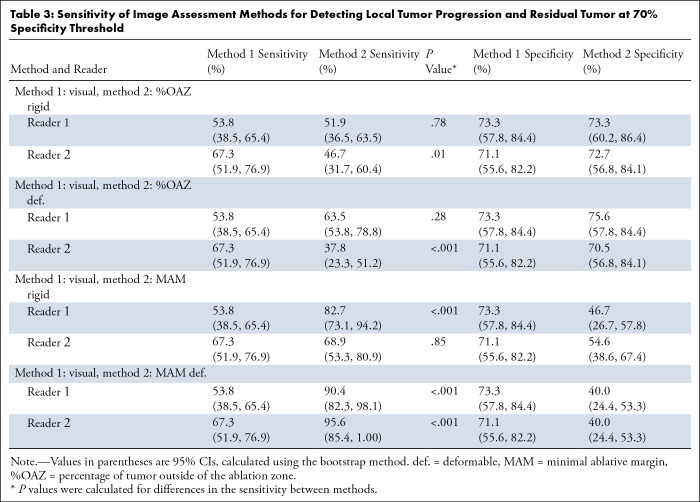
Sensitivity of Image Assessment Methods for Detecting Local Tumor Progression and Residual Tumor at 70% Specificity Threshold

### Interobserver and Intraobserver Agreement

Inter- and intraobserver agreement is summarized in Table 4. Intraobserver agreement was almost perfect for all quantitative metrics, with Gwet AC1 of 0.80 (95% CI: 0.70, 0.90) and 0.92 (95% CI: 0.86, 0.99) for MAM using rigid and deformable registration, respectively, and ICC of 0.86 (95% CI: 0.80, 0.91) and 0.95 (95% CI: 0.93, 0.97) for %OAZ using rigid and deformable registration, respectively. Interobserver agreement was moderate for MAM with rigid registration, with Gwet AC1 of 0.43 (95% CI: 0.27, 0.59), and substantial for deformable registration, with Gwet AC1 of 0.67 (95% CI: 0.54, 0.80). %OAZ was moderate for both rigid and deformable registration, where ICC was 0.58 (95% CI: 0.43, 0.71) and 0.41 (95% CI: 0.23, 0.57), respectively. Interobserver agreement was significantly higher for MAM with deformable registration than visual assessment (Gwet AC1 0.67 vs 0.18, *P* < .001) and for MAM with deformable versus rigid registration (Gwet AC1 0.67 vs 0.43, *P* = .006).

**Table 4:**
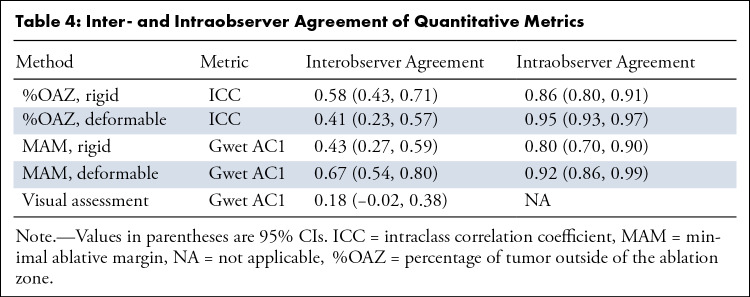
Inter- and Intraobserver Agreement of Quantitative Metrics

## Discussion

Motivated by the limited global availability of contrast-enhanced CT in ablation procedures, in this study we aimed to rigorously validate several potential quantitative imaging biomarkers for classifying successful ablation of CRLM using pre- and postprocedure images with extended intervals between scans. These quantitative assessments were benchmarked against visual image inspection. Our approach adhered to the principles of the quantitative imaging biomarker development roadmap, including both technical validation (repeatability and reproducibility) and clinical validation (assessment of tumor outcomes).

Visual assessment demonstrated poor interobserver reproducibility (Gwet AC1, 0.18), which underscores the need for more reliable imaging biomarkers that support patient management by identifying tumors at higher risk of recurrence and enabling intensive surveillance and earlier retreatment where necessary. Our findings indicate that quantitative methods, for example MAM, offered improved reliability versus visual assessment (Gwet AC1, 0.67 vs 0.18; *P* < .001 for interobserver agreement) and higher sensitivity (>90% sensitivity at 40% specificity), albeit with trade-offs in specificity and increased time requirements.

Performance of our quantitative margin assessment metrics (AUC between 0.57 and 0.76) was lower than in the published literature concerning use of immediate postablation contrast-enhanced CT imaging, where AUCs between 0.77 and greater than 0.9 (23,24) have been reported. A pertinent recent study demonstrated superior performance where registration-based ablation confirmation was performed using intraprocedural imaging (AUC, 0.89) versus initial follow-up CT (AUC, 0.66) (25). This discrepancy in performance is likely due to ablation zone involution over time, which deformable surface-based ablation confirmation does not account for (5). This limitation results in falsely small ablation margins and thus a tendency to “overcall” incomplete ablation, explaining our high proportion of cases whereby incomplete ablation was called (approximately 70%, and out of proportion with residual tumor and LTP), thus limiting specificity. Together with the existing literature, our results emphasize the critical importance of acquiring high-quality, immediate intraprocedural contrast-enhanced CT images to optimally assess ablation success. Alternatively, further work is required to optimize biomarkers where pre- and postprocedure imaging is used, for example, acquisition of images at standard follow-up times, negative values of MAM, or using models that account for ablation zone involution.

Another study (26) reported much higher performance of three-dimensional ablation confirmation using pre- and postprocedural follow-up imaging for discriminating LTP (AUC, 0.89). However, the authors excluded cases in which ablation confirmation was deemed inadequate, leading to selection bias, and their quantitative analysis was not performed blinded to reference standard.

Our finding of high sensitivity with quantitative metrics is similar to that of Vasiniotis Kamarinos et al (13) who reported a sensitivity and specificity of 93% and 42%, respectively, when using MAM for assessment of LTP. High sensitivity is particularly advantageous for screening and could potentially be used to augment the more specific technique of visual inspection (>70% specificity at >50% specificity).

Differences in interobserver agreement of quantitative imaging biomarkers, for example MAM with deformable versus rigid registration (Gwet AC1, 0.67 vs 0.43; *P* = .006), indicate that not all quantitative ablation margin biomarkers are equally effective. Further research must therefore investigate multiple biomarkers, including combinations, to enhance assessment accuracy. For example, one advantage of the %OAZ over MAM is that when tumors are located at the liver periphery and adjacent to blood vessels, completely ablated tumor will yield %OAZ of 0 (ie, complete coverage), whereas MAM of 0 mm is more difficult to interpret in these settings.

Most of the existing three-dimensional techniques available for clinical use rely on either rigid or intensity-based deformable ablation confirmation. However, both are susceptible to registration errors resulting from liver deformity caused by patient positioning, breathing, placement of the ablation applicator, hydrodissection, and tissue contraction associated with the ablation process. Alternative image registration methods, such as biomechanical deformable image registration, have shown utility in ablative margin assessment (27) and can account for differences in liver position and breathing (28).

Compared with rigid registration, liver deformation has the potential to mitigate against changes in liver orientation that occur when imaging at multiple time points. However, data regarding reliability are lacking. In a systematic review of 29 studies (29) assessing software-based assessment after thermal ablation of liver tumors, only six studies reported interobserver reproducibility; five of these studies included MAM, and only two included CRLM. None included intraobserver agreement assessment, nor comparison with visual assessment, meaning these emerging techniques require further validation (13,24,26,30).

An interobserver reproducibility assessment was performed in only one study of patients (*n* = 18) with CRLM (31). The authors found perfect agreement for ablation completeness and substantial agreement (κ = 0.723) for individual thresholds (≤0 mm, 1–5 mm, ≥5 mm).

In the present study, many tumors demonstrated LTP or residual tumor (54% with median time to censorship of 25 months), which is higher than in some previous cohorts (24) but similar to the findings of Kaye et al (26) (58% within 24 months). The reasons for this are multifactorial. First, our cohort included cases from relatively early stages of global experience with MWA, when the importance of generous margins was not widely recognized. This is particularly relevant to our practice where tumors often exhibit aggressive biologic behavior and resistance to treatment after multiple lines of systemic therapy. For example, treated tumors may leave behind an “imaging-invisible” but viable footprint of tumor cells. Second, tumors were typically small and technically challenging to ablate. Furthermore, we did not routinely perform contrast-enhanced CT during ablation, which has since shown to improve outcomes (32). Despite this, use of intraprocedural contrast-enhanced CT is still not ubiquitous when performing ablation and assessing technical success (14) and not always available for US-guided and intraoperative ablation, whereas diagnostic pre- and postablation CT are standard practice. We have since enhanced our practice to include robotic guidance (33), vacuum immobilization, high-frequency jet ventilation, contrast-enhanced CT at time of ablation, software-based margin assessment, and immediate reablation when margins are insufficient. None of the tumors included in the study cohort were treated using this new practice. As such, it is likely that our oncologic outcomes have since improved, in line with the temporal improvement that was shown in the AmCORE registry (34).

Strengths of this study include a real-world cohort, for which all tumors were included regardless of registration error, leading to more realistic performance estimates. Second, we have rigorously evaluated two different imaging biomarkers derived using both rigid and deformable ablation confirmation workflows versus visual assessment according to the imaging biomarker development roadmap, which is critical for clinical translation of valid biomarkers (17).

Limitations of the study included its retrospective nature and being conducted at a single specialized center, where case complexity is unlikely to reflect wider ablation practice. Additionally, sample size was determined by availability of datasets. As with any imaging-based reference standard, it is also possible that some tumors abutting ablation zones represented new tumors rather than LTP. Not all tumors had at least 1 year of imaging follow-up, as some patients returned to their local centers, which may have impacted LTP incidence. Additionally, subcapsular and perivascular tumors were analyzed the same way, which may impact assessment of MAM given that tumors do not usually grow outside the liver or into adjacent vessels.

Further work is needed to expand the range of quantitative metrics available for assessing ablation success. Advanced analysis techniques, such as machine learning with separate development and test cohorts, could provide more robust outcome assessments. Automation of these workflows would also streamline processes, making them more feasible for clinical use. Additionally, adjustments to account for ablation zone involution over time could enhance accuracy. Incorporating ablation confirmation techniques that evaluate factors beyond simple image intensity, such as shape and density, may provide more comprehensive evaluations. To further increase reliability, interobserver agreement could be enhanced through standardized training protocols. Finally, reducing the time required to process the deformable workflow would facilitate its clinical implementation.

In summary, quantitative ablation margin metrics derived from software-assisted ablation confirmation techniques using pre- and postprocedure contrast-enhanced CT imaging demonstrate substantial promise as more reliable imaging biomarkers for residual or recurrent tumor following thermal ablation of colorectal cancer liver metastases, compared with visual assessment.
